# Resilience phenotypes derived from an active inference account of allostasis

**DOI:** 10.3389/fnbeh.2025.1524722

**Published:** 2025-05-09

**Authors:** Laura A. Harrison, Antonio J. Gracias, Karl J. Friston, J. Galen Buckwalter

**Affiliations:** ^1^Valor Institute for Neuroscience and Decision Making, Chicago, IL, United States; ^2^Queen Square Institute of Neurology, University College London, London, United Kingdom; ^3^VERSES AI Research Lab, Los Angeles, CA, United States

**Keywords:** resilience, allostasis, active inference, free energy principle, phenotype, enactive niche

## Abstract

Within a theoretical framework of enactive allostasis, we explore active inference strategies for minimizing surprise to achieve resilience in dynamic environments. While individual differences and extrinsic protective factors traditionally account for variability in resilience trajectories following stressor exposure, the enactive model emphasizes the importance of the physical and social environment, specifically the “enactive niche,” which is both shaped by and impacts organisms living in it, accounting for variable success in allostatic prediction and accommodation. Enactive allostasis infers or predicts states of the world to minimize surprise and maintain regulation after surprise, i.e., resilience. Action policies are selected in accordance with the inferred state of a dynamic environment; those actions concurrently shape one’s environment, buffering against current and potential stressors. Through such inferential construction, multiple potential solutions exist for achieving stability within one’s enactive niche. Spanning a range of adaptive resilience strategies, we propose four phenotypes—fragile, durable, resilient, and pro-entropic (PE)—each characterized by a constellation of genetic, epigenetic, developmental, experiential, and environmental factors. Biological regulatory outcomes range from allostatic (over)load in the fragile and durable phenotypes, to allostatic recovery in resilience, and theoretically to increasing allostatic accommodation or “growth” in the proposed PE phenotype. Awareness distinguishes phenotypes by minimizing allostatically demanding surprise and engenders the cognitive and behavioral flexibility empirically associated with resilience. We further propose a role for awareness in proactively shaping one’s enactive niche to further minimize surprise. We conclude by exploring the mechanisms of phenotypic plasticity which may bolster individual resilience.

## 1 Introduction

The term “resilience” has been aptly described as polysemous (Miller et al., 2022). However imprecise its meaning, its scientific and social relevance is widely evident. All-cause mortality and morbidity associated with chronic and acute stress are mounting public health concerns (Magruder et al., 2016). Diseases associated with chronic stress (heart, cancer, stroke, Alzheimer’s, diabetes) are consistently among the top 10 causes of death in the US (Curtin et al., 2024a, 2024b; Heron, 2021). Persistent stress without a defining traumatic event, such as a toxic work environment, negatively affects well-being (Friedline et al., 2021), hurts workplace performance (Bui et al., 2021), and increases burnout (Yates, 2020). The mental health impact of natural disasters was consistently negative in a recent meta-analysis (Keya et al., 2023). Meanwhile, combat exposure (Bricknell et al., 2020), sexual assault (Dworkin, 2020), and motor vehicle accidents (Marasini et al., 2022) are a few of the acute adverse life events with negative long-term mental health effects. Despite the ubiquity of stress and trauma and their burden, resilience to stress is remarkably common and may be the default response to adversity (Bonanno, 2021).

The scientific conceptualization of resilience has evolved over time. Early work focused on innate traits (Kobasa, 1979; Kobasa and Puccetti, 1983), a perspective soon challenged when resilient outcomes, mostly defined as the absence of psychopathology, were associated with malleable behaviors, psychological constructs (Agaibi and Wilson, 2005; Werner, 1999), and social resources (Zautra, 2013). More recent formulations continue to focus on good mental health after adversity as the outcome of resilience but place its maintenance in the context of developmental, multisystem networks including communities (Masten et al., 2021), cognitive mechanisms (Kalisch et al., 2015), and regulatory flexibility (Bonanno et al., 2023, 2024).

Building on the seminal work of Bonanno et al. (2011) that identified prototypical resilience trajectories after a potentially traumatic event, Bonanno et al. (2023) recently describe *prospective* trajectories (Bonanno et al., 2023) when previous states impact one’s resilience trajectory. In Kalisch et al.’s. (2024) formulation, “stress reactivity” is indexed at frequent intervals observing resactivity under varying amounts of stress, allowing for identification of causal resilience mechanisms and processes. In both accounts, resilience is understood as an ongoing, dynamic, complex, biopsychosocial process. Building on this foundation, we propose a theoretical framework for the mechanisms that control this dynamic process of resilience. In so doing, we will identify phenotypes that are aligned with the trajectories of resilience previously identified (Bonanno et al., 2023), and advance an additional phenotype, namely, pro-entropic resilience.

Resilience cannot be described apart from a stressor, which is defined as “a stimulus or situation that elicits a stress response” (Kalisch et al., 2021). Despite the seeming circularity of this definition, we find it offers a path forward in discussing resilience. We accept the necessary link between resilience and the stress response, based on an understanding that the stress response includes a reaction to a stimulus and the potential for accommodation.

We argue this two-stage formulation of a stress response is consistent with the theory of allostasis. Allostasis was defined by Sterling and Eyer (1988) as “stability through change,” allowing organisms to maintain stable regulatory systems, e.g., homeostatic systems such as osmosis, within dynamic environments. Prediction is a requirement for the brain to maintain regulatory efficiency during change (Sterling, 2012). Allostasis is the predictive interaction of a multisystem biological organism with their environment (Ungar and Theron, 2020). In what follows, we advance an enactive formulation of allostasis by considering it within the framework of active inference. In this context, we use the term enactive allostasis to foreground the predictive nature of allostasis and its dependency on the niche. When framed as an enactive process, allostasis rests on all of the processes entailed by (en)active inference—to be defined throughout—allowing for a formal conceptualization of resilient phenotypes.

Developed from the free energy principle (FEP)—an information-theoretic principle that provides an optimization target by which self-organizing agents maintain an equilibrium (steady-state) exchange within their environment through the minimization of surprise or uncertainty (Friston et al., 2006; Friston, 2010)—our model reframes resilience within this principle of self-organization. In active inference,^1^ the brain essentially acts as a (Bayesian) prediction-processing network, where prior beliefs, or predictive models, are updated with new sensory information to form posterior beliefs through perceptual inference. Importantly, the brain not only reduces uncertainty through perception, it also actively samples the world as an embodied agent (Friston et al., 2010) and enacts policies to effect predictions (Ramstead et al., 2020). Further, active inference presupposes the inferential development of sentient (sense-making) processes which places awareness into our enactive model of allostasis.

The totality of all biopsychosocial systems within the environment in which an allostatic prediction is made is what we call an “enactive niche.” While our focus is on resiliency in active inference, we find it to be phenotypic within the entire spectrum of allostatic profiles possible in an enactive niche. We propose that a phenotype’s resilience is determined by the ability to use predictive processing to actively engage with, adapt to, and proactively shape the enactive niche, all for the allostatic accommodation necessary to reduce future surprise. In this framework, resilience results from features of the niche, broadly defined, as well as one’s predictive strategy for exchanging with it. It is this phenotypic characterization of resilience that complements the existing understanding of resilience as the interaction of dynamic biopsychosocial systems. Our model accommodates phenotypes that reflect the tendency of complex systems to revisit characteristic states (Ramstead et al., 2018), as well as plasticity within phenotypes (Murren et al., 2015). This provides a framework within which to understand individual differences in resilience. Further, within this paradigm, phenotypic plasticity, either toward or away from resilience, is explainable as an enactive process, one of bi-directional influence between the organism and the environment. We focus on the role that awareness—i.e., sensory processing, interoceptive inference, and higher level, “top down” processing—plays in allowing for growth of the individual, as an allostatic system, as part of an enactive niche to highlight strategies for individualized change.

This paper aims to offer a tractable and unifying multidisciplinary model of resilient phenotypes defined using the construct of active inference. While the current outcomes and trajectory approaches to resilience conform to the concepts of the enactive niche, the proposed approach has the advantage of placing resilience within the full spectrum of allostatic responses that are inextricably linked with awareness and the status of the organism in its physical and social environments. Thus, this formulation considers resilient factors, mechanisms, processes and outcomes in a single model, where phenotypes emerge from the entirety of the predictions made across all these variables that are part of the process of enactive allostasis. This approach is also in alignment with the NIH Concept Model (Brown et al., 2023), which considers the type of stressor, the entire system across molecular, physiologic, psychosocial, and environment/community levels and the system’s response over time.

## 2 Resilience

This section explores the concept of resilience, its definitions, and contributing factors.

### 2.1 Resilience defined

Some degree of resilience is putatively accepted to be innate in the human response to adversity (Bonanno, 2021). Early work defined resilience as the absence of psychopathology, often PTSD (Agaibi and Wilson, 2005) after trauma. Traumatic events are common worldwide with exposure estimates ranging as high as 70% (Benjet et al., 2016). Disentangling the prevalence of long-term effects on mental and physical health is not yet feasible but lifetime estimates of PTSD are around 8% (Gradus, 2017). However, the classic 32-year longitudinal study of Kauaian children who had experienced adverse developmental conditions found one out of three grew into competent, confident, and caring adults (Werner, 1999) suggesting a profound effect of developmental chronic stress, along with remarkable resilience. This work led to a broader notion of resilience as “good survival,” or adaptation to and recovery from stress (Bonanno and Mancini, 2012; Masten, 2001). In good survival, some may even be positively changed by this adaptive process (Boden et al., 2014), through successful negotiation and management of the stressors experienced across the lifespan (Windle, 2011) to result in what has been described as post-traumatic growth and psychosocial gains from adversity (Johnson and Boals, 2015; Mancini, 2019).

#### 2.1.1 Biopsychosocial contributors to resilience

Support for human resilience exists across multiple biopsychosocial systems. We provide a high-level review of such contributors.

##### 2.1.1.1 Genetic and epigenetic effects

Individual differences in allostatic systems and their signaling pathways may be borne of genetics, epigenetics, and experience. The genetic effects on resilience have been described as undeniable despite inconclusiveness about underlying mechanisms (Maul et al., 2020). Niitsu et al. (2019) review the genetics of the psychological manifestation of resilience and report six genes involved in numerous biopsychosocial components of resilience, including neuroplasticity, emotional regulation and social bonding. This is consistent with the complexity operationalizing the genetics of all behavioral factors (Madole and Harden, 2023).

Epigenetic modifications change gene expression without altering the DNA sequence, with gene expression shown to be altered by both positive and negative environmental factors (Schiele and Domschke, 2018) and to distinguish between vulnerable and resilient when confronted with stressors (Smeeth et al., 2021). Epigenetic resilience has been proposed to be inheritable, generational, and impacted by developmental challenge and protection (*ibid*).

##### 2.1.1.2 Neuropsychological contributors to resilience

The recognition of individual differences as relevant to resilience stems largely from the work of Lazarus. Recognizing variance in how individuals interpreted—i.e., appraised—the same stressful situation, he attributed these differences to motivational and cognitive variables that intervened between a stressor and affective response (Lazarus and Eriksen, 1952).

Since this pivotal work, emotion regulation and appraisal has been central in the conceptualization of resilience (Tabibnia, 2020). Appraisal is most often discussed in the context of changing emotions or the usual trajectory of emotions, i.e., mood (Uusberg et al., 2019). We will not explore emotion itself but accept the definition by Damasio as “complex programs of actions triggered by the presence of certain stimuli, external to the body or from within the body, when such stimuli activate certain neural systems” (Damasio, 2011). While appraisal is a top-down process, i.e., using awareness to alter the emotion, the broader category of emotional regulation employs a variety of strategies (Naragon-Gainey et al., 2017) including some that do not directly involve the use of awareness of the emotion, such as exercise (Bernstein and McNally, 2018) or meditation (Menezes and Bizarro, 2015).

There is widespread support for the idea that attention becomes focused on the source of stress with a concomitant loss of attention to other aspects of the environment (Chajut and Algom, 2003; Schwabe et al., 2013). Indeed, stress presents another seeming paradox, with distress impairing learning and performance, and the correct amount of stress, or eustress, improving performance. Compounding this effect, researchers have recognized that beliefs about stress shape its impact on learning (Rudland et al., 2020) and health (Keller et al., 2012). This relates to the bias that occurs under stress to rely on habit (i.e., exploit known solutions), rather than flexibly explore options that optimize goal-directed decision-making (Yu, 2016), essential to convert distress to eustress.

Stress negatively impacts cognitive flexibility (Alexander et al., 2007; Goldfarb et al., 2017; Seehagen et al., 2015), more so in men than women (Knauft et al., 2021; Shields et al., 2016). Flexible direction of attention is key for cognitive and behavioral flexibility. Cognitive flexibility is required to avoid undue bias in decision-making, which we argue increases the likelihood of avoiding allostatically demanding surprise during environmental change. Bonanno et al. (2024) place psychological flexibility as central to stress accommodation. Behavioral flexibility is also adaptive and linked with resilience (Iacoviello and Charney, 2020); an effect we believe builds on cognitive and psychological flexibility. Cognitive flexibility is linked to the locus coeruleus (LC) (Sales et al., 2019), which modulates arousal and whose functional connectivity with the salience network modulates attention (Neal et al., 2023). The LC has long been studied as part of the brain’s alerting and stimulus detection system. The LC is the major source of the catecholamine norepinephrine in the brain (Sales et al., 2019). Phasic responses are short, high frequency activations associated with behaviorally relevant, salient stimuli, which facilitate a shift to short-term behavioral planning. Meanwhile, extremely high levels of tonic LC firing, linked to arousal, are associated with behavioral variability and stochastic decision making and a shift from exploitation to exploration strategies (Morris et al., 2020), as well as inhibition of prefrontal functions (Krystal and Neumeister, 2009). Chronic stress has been shown to increase responsivity of LC neurons to excitatory stimuli (Morris et al., 2020) with chronic stress causing greater cholinergic reactivity (Southwick et al., 1999). Acute stress has also been suggested to persistently alter LC functioning (Borodovitsyna et al., 2018). More recent formulations within predictive processing have suggested LC firing is a correlate of prediction error when inferring states for action planning (Sales et al., 2019).

The catecholamine, dopamine, is also released in response to stress and related to predictive processing in the brain. Widely studied for its role in motivation and reward systems (Wise, 2004), its direct role in the stress response is recognized but not well understood. Dopamine and norepinephrine are implicated in enhanced vigilance, focused attention, and increased SNS activity, raising blood pressure and cardiac output (Beauchaine, 2009). It has been argued that dopamine encodes the precision or certainty afforded to responses or plans (FitzGerald et al., 2015; Friston et al., 2014; Friston, 2009; Schwartenbeck et al., 2015) rather than simply reward prediction error.

Emotional regulation unites the neuropsychology of resilience (Hunter et al., 2018), summarized in a tripartite structure: (1) down-regulating the negative through appraisal; (2) up-regulating the positive through social connections, flexibility and a positive sense of self; and (3) transcending the self through spirituality and experiences of joy and awe (Tabibnia, 2020). We carry this working framework forward in our proposed resilience phenotypes.

##### 2.1.1.3 Social contributors to resilience

As a social species (McCall and Singer, 2012), social stress and support inversely impact resilience. Adverse social interactions (e.g., interpersonal violence, neglect) are key risk factors for stress disorders, while social protective factors (social support and emotional connection) are associated with resilience. Generally, positive social ties with other individuals, groups, or the larger community offer social support (Lin et al., 1979). Acute stress response, indexed by increases in heart rate, blood pressure, and cortisol, decreased in individuals accompanied by a support companion relative to those who faced a stressful task unaccompanied (Kamarck et al., 1995; Kirschbaum et al., 1995). Building on this, a recent study showed that active rather than passive support most effectively diminishes the subjective experience of and physiological response to pain (Mazza et al., 2023).

A recent process explaining relationships’ contribution to individual allostasis has been theorized under the rubric of social allostasis (Saxbe et al., 2020). Recognizing the conflicting effects that relationships can have on allostasis, the authors hypothesize relationships serve as regulators where groups strive for homeostatic balance and individuals work together to maintain group emotional and behavioral baselines.

### 2.2 Current theories of resilience

Biopsychosocial systems research has demonstrated that resilient individuals have identifiable traits and practices, or resilience factors that are shaped by genetics (Feder et al., 2009), epigenetics (Smeeth et al., 2021; Zannas and West, 2014), culture, social resources (Hobföll, 1989, 2001), and life circumstances (Armbruster et al., 2012). Some argue that stress resilience is distinct from recovery from trauma (Richter-Levin and Sandi, 2021), although others argue that the same practices that help resilient individuals overcome significant adversity provide transferrable protection to coping with stress (Fletcher and Sarkar, 2013). We argue that enactive allostasis can explain resilient processes and outcomes associated with both trauma and with chronic stress.

While relatively static trait and malleable contextual resilience factors have been combined to reliably predict the likelihood of resilience outcomes at the group level, resilience outcomes are very difficult to predict in individuals. This contextual dependence has been labeled the “resilience paradox” (Bonanno, 2021).

Similar to the resilience trajectories introduced above (Bonanno, 2004; Bonanno et al., 2011, 2023; Galatzer-Levy et al., 2018), Kalisch et al. (2024) provide a longitudinal model to enhance the valid measurement of resilient processes and resilient outcomes (Kalisch et al., 2021). Dubbed the Frequent Stressor and Mental Health Monitoring (FRESHMO) paradigm, the ratio of mental health reactivity to stress exposure, termed “stress reactivity,” is calculated across time.

Bonanno’s earlier work identifying four prototypical trajectories of adjustment following potentially traumatic events (Bonanno, 2004; Bonanno et al., 2011) also comports with the notion of allostatic phenotypes. A meta-analysis (Galatzer-Levy et al., 2018) confirmed those trajectories: (1) no dysfunction following the event; (2) immediate dysfunction with gradual recovery; (3) a delayed trajectory with dysfunction increasing over time; and (4) an emerging chronic level of dysfunction. Bridging work on trajectories and FRESHMO, we propose the resilient outcome of individuals reflects the accuracy of the brain’s predictive models that undergird regulatory control during and after stress and/or adversity. i.e., enactive allostasis. Prediction includes all the biopsychosocial systems associated with resilience in a complex, dynamic model from which phenotypes emerge. These phenotypes allow for the application of our theoretical approach to individuals.

## 3 Allostasis

To survive, all biological systems must maintain physiological stability. Physiologist Cannon (1929) labeled the drive to maintain a stable internal milieu (e.g., temperature, blood pressure, blood glucose) “homeostasis” and proposed it operated as an automatic negative feedback model requiring coordination of multiple local organs. Adjustments were thought to be made in response to negative internal conditions with the goal of maintaining near constant internal conditions (*ibid*).

The concept of allostasis (‘stability through change’) was first to place homeostatic regulation under control of the central nervous system (Sterling and Eyer, 1988). For the brain to efficiently maintain regulation during change it must use prediction (Sterling, 2012). As noted above, resilience requires a stress response that entails a physiological reaction and accommodation. Allostasis further suggests that the stress response is predictive, with systems changing from baseline conditions to accommodate perceived challenges. Allostatic predictions are effected primarily through the autonomic nervous system (ANS) and the hypothalamic–pituitary–adrenal (HPA), but also through a host of metabolic, inflammatory, neuromodulatory and other systems, Korte et al. (2005) allowing for accommodation to perceived stress, both physical and psychological (Sterling and Eyer, 1988).

The degree of the reactive response and the completeness of the recovery have the potential to alter allostatic baselines (Schulkin and Sterling, 2019; Sterling, 2012; Sterling, 2004; Sterling and Eyer, 1988). Allostatic predictions may be subthreshold and thus not result in physiological change. However, the effectiveness of an allostatic prediction is evident in the stability, or return to baseline, of the systems that are regulated in response to the prediction McEwen identified incomplete accommodation as allostatic load (AL), the cumulative physiological and psychological burden (“wear and tear on the brain and body”) of chronically adapting to stress (McEwen, 1998; Seeman et al., 1997). Further, when allostasis-induced neurophysiological changes exceed the capacity of a person to function in the short-term, allostatic *overload* occurs (Fava et al., 2019). This is clinimetrically defined by overwhelming stress resulting in physiological (Offidani and Ruini, 2012), affective, and social disruptions.

Chrousos and Agorastos have provided helpful distinctions between allostatic outcomes by differentiating the motivating effect of *eustress* from the harmful impact of *distress*, arguing the former leads to *hyperstasis* (“higher/better) state,” in which one’s ability to maintain homeostasis is improved, while the latter leads to *cacostasis* (“bad state”) or dyshomeostasis (Chrousos, 2009; Agorastos and Chrousos, 2022). They further identified distress and cacostasis as defining a vulnerable phenotype, which we find aligned to our framing of resilient phenotypes, specifically the fragile phenotype. The distinction drawn between eustress and distress is helpful but one we see as implicit in allostasis when considering the degree of accommodation. For simplicity and continuity with the bulk of the literature, we discuss the stress response as an allostatic prediction to a challenge. This prediction leads to a physiological reaction and accommodation. With complete accommodation, allostasis—as historically defined (stability through change)—ensues, which read as resilience. We distinguish between this allostasis with recovery, which includes eustress, and incomplete allostatic recovery including allostatic load and allostatic overload. We will also explore the possibility of improvement following an allostatic prediction or “allostatic growth,” as a unique type of resilience. Further, we recognize the possibility of a range of allostatic responses and their short- and long-term effects on outcomes. Indeed, within this context, the tendency of complex systems to revisit characteristic states, or phenotypes, becomes apparent (Ramstead et al., 2018) when considering the range of possible patterns of allostatic reactivity and accommodation.

Briefly,^2^ the ANS is regulated by a complex neural network that responds to both internal and external demands. Its basic structure provides counter regulatory mechanisms to activate and recover from allostatic reactions. The ANS contains two concurrently acting systems, the sympathetic (SNS) and parasympathetic nervous systems (PNS) (LeBouef et al., 2023). Activation of the SNS prepares the body for action while the PNS returns systems to a more sustainable baseline. Each consists of discrete functional pathways that may be activated independent of one another or in the specific pattern needed to maintain homeostasis given the organism’s resources and the current environment.

The HPA has been called the central driver of allostasis through the glucocorticoid, cortisol (de Kloet and Joëls, 2023). Cortisol controls allostasis by a receptor-mediated on-and-off switch, which regulates the organism to provide the energy required to maintain homeostasis. The presence of two distinct receptors, the mineralocorticoid (MR) and glucocorticoid receptors (GR), each with different affinities for cortisol, allow for organism-specific patterns of allostatic activation and accommodation.

The lifelong impact of developmental trauma and stress suggests critical periods for brain exposure to HPA activity (Agorastos and Chrousos, 2022). Early trauma, commonly referred to as Adverse Childhood Experiences (ACE), show long-term effects on numerous physical and mental health outcomes (Sheffler et al., 2020; Wittchen et al., 2011). Results have been equivocal on the long-term impact of ACE on adult cortisol reactivity, although recent meta-analyses concluded there was a blunting effect on cortisol release among adults with ACEs during mental stress (Bunea et al., 2017) and on cortisol and cardiovascular reactivity during social stress (Brindle et al., 2022).

The adrenal androgen released in response to stress, dehydroepiandrosterone (DHEA), and its sulphated ester (DHEA-S), provides a mechanism that supports allostatic recovery. It acts as a glucocorticoid antagonist to protect the brain, particularly the hippocampus, from negative effects of cortisol (Kamin and Kertes, 2017). It has numerous neuroprotective mechanisms (Maggio et al., 2015) and is associated with improved emotional regulation (Sripada et al., 2013).

While the allostatic system is replete with mechanisms that counteract the potential for stress to result in long-term negative consequences (Osório et al., 2017), oxytocin may have a unique role in resilience. The oxytocin system provides the neurohormonal substrate for parental, romantic, and filial attachment (Feldman, 2012). The role of oxytocin in attachment and stress modulation supports the concept of “affiliative resilience” (Feldman, 2020). Social affiliation has long been recognized to have allostatic implications (Sterling, 2012).

### 3.1 Allostatic prediction and resilience

Fundamentally, prediction both anticipates events that may occur and prepares the system to maintain regulation before the need arises. Predictive allostasis reduces error, matches the capacities of different response components, shares resources among systems to reserve overall capacity, and integrates past errors to improve future predictions (Sterling, 2012). In prediction, the brain develops models based on prior experience and traits to allocate resources needed to react to stress. Successful allostatic predictions not only indicate an ability to predict the internal demands needed to respond to an environmental change, e.g., fight or flight, but it also suggests the subsequent allostatic accommodation, e.g., rest and recovery. Allostatic predictions can result in allostatic responses that ultimately lead to chronic homeostatic disruption—e.g., the association of diabetes with AL (Steptoe et al., 2014). It is equally plausible to argue predictive allostasis can maintain and potentially enhance the efficiency of the neuropsychobiological systems that maintain homeostasis across time and environmental change.

As elegantly evidenced in the regulation of cortisol (de Kloet and Joëls, 2023), stress responses demonstrate that with every activation there is a possible recovery mechanism. Stress accommodation is situational, determined by interaction with the broad physical and affiliative environment, shaped by factors occurring across variable time courses and realized across all levels of neurobiological expression, genetic to behavioral. Thus, we argue that allostatic predictions are enactive, informed by both the person and their environment, where we use the terms enactive and predictive allostasis interchangeably. Within this context, a broad range of individual differences in predictive allostasis is observed, which are presented as phenotypes.

## 4 Free energy and active inference: a review

The FEP is an information-theoretic construct that provides an optimization target, the minimization of surprise, by which self-organizing agents maintain a (far from equilibrium) steady state within their dynamic environment (Friston, 2010, 2012). Minimizing the long-term average of surprise—reflecting the divergence between the organisms’ predicted and observed sensory exchanges with the world—allows the organism to minimize the entropy of sensory states (Friston, 2010) and, implicitly, disorder in the sensorium (Ramstead et al., 2018). This highlights a core distinction between homeostasis and allostasis—made under the FEP—that allostasis requires predictive models of one’s agency in relation to the external environment (Constant et al., 2018; Ramstead et al., 2018, 2021). For clarification, we do not distinguish between the terms used to refer to this phenomenal sense of self, such as self-consciousness, cognition, awareness, etc. and rather arbitrarily use the term awareness.

Beliefs, or predictive models, are evaluated for accuracy against relevant external and internal sensory inputs; namely, the differences between predictions and sensory inputs; i.e., prediction errors (Friston, 2010) in perceptual inference. Precision highlights the important role of selecting sensory inputs for predictive processing. Without complete precision, or reliability, beliefs are updated under some degree of uncertainty, with some expectation of surprise. This draws a distinction between expected and unexpected surprises that we relate to awareness. Beliefs about intended states of being are enacted through *policies,* or plans of action (c.f., ideomotor theory). Policies are inferential and selected to alter the environment in ways that will minimize surprise by changing sensory inputs to better align with predictive models (Pezzulo et al., 2018). For instance, we pull down our visor while driving to deflect the sun. Just as this policy allows us to avoid the unwanted surprise of driving blindly, a similar selection of policies is used to alter our awareness, including our sense of self, again with the goal of decreasing surprise.

Belief updating through active inference samples the world, both proprioceptively and exteroceptively, as an embodied agent (Friston et al., 2010). Those inferences that alter sensory input—i.e., change exteroceptive and or proprioceptive inferences through action—include inference as the basis of policy selection; namely, inferring what one is likely to do next and then engaging motor systems and autonomic reflexes to realize the resultant predictions (Ramstead et al., 2020). This places perceptual inference under active inference, in the sense that inferred states of the world, i.e., perceptual inferences, are used to inform beliefs about acting as an agent on that world. This synergism between action and perception optimizes predictive models to maintain homeostasis (Badcock et al., 2019) through enhanced allostatic predictions. We further agree with Ramstead (ibid) that the inferential process is quintessentially enactive: each generative model couples the individual with their social and physical environment, forming an enactive niche.

Our brains constantly predict our internal states in the context of our environment and where we are in it: *exteroceptive* predictions are measured against external sensations from the extrapersonal environment; *proprioceptive* predictions are measured from the sensation of the body moving in the environment; and *interoceptive* predictions are measured against internal sensations from the body. Interoception is the mental process of inferring the internal status of our regulatory systems, both homeostatic (Duquette, 2017; Fotopoulou and Tsakiris, 2017) and allostatic (Corcoran and Hohwy, 2018). The role of interoception takes priority in enactive allostasis, as well as in affective processing (Quadt et al., 2022) and resilience (Haase et al., 2016). Active inferences are made with priors that consider the accuracy of every prior relevant inference, with current inferences selected that have the highest probability of minimizing surprise, i.e., uncertainty or entropy.

Active inference further presupposes the inferential development of sentient (sense-making) processes, including perception (Parr et al., 2019), interoception (Corcoran and Hohwy, 2018), up to and including the deep temporal models that underwrite epistemic awareness or sense of self (Friston et al., 2016; Friston K. J., 2018; Seth and Friston, 2016; Vilas et al., 2022). Awareness or basic sentience has evolved to minimize expected surprise, or uncertainty, over time (Nave et al., 2022). The possibility that awareness contributes to the organism’s inferential process in ways that alter allostatic systems drives our interest in formalizing a role for awareness in active inference, which in turn relates to our framework for resilience. Just as our belief in our actions and plans becomes strengthened with precision, so does our belief in ourselves (Friston et al., 2015), e.g., as capable and effective, or ineffective and subject to external forces, or somewhere in between.

Central to our understanding of active inference is that awareness is an outcome of a policy selection that reduces expected surprise (Nave et al., 2022). Predicting and realizing sensory data—in a complex physical and social environment—requires integration across interoceptive, proprioceptive and exteroceptive sensory modalities, under deep temporal models, which underwrites a stable, ideally positive sense of self, mental time travel, and cognitive flexibility. The implicit role for awareness in allostatic prediction also necessitates aspects of perception that can increase AL. Indeed, stress has been formulated as an increase in expected free energy (EFE)—i.e., uncertainty (Ueltzhöffer et al., 2021)—in an aware decision-making context.

Enactive allostasis happens within one’s established phenotype. A host of genetic, epigenetic, and developmental influences define the phenotypic states that the individual returns to with high frequency. In a predictive allostatic context, returning to phenotypic, i.e., preferred, characteristic, or unsurprising sensory states (Arnaldo et al., 2022), underwrites allostatic accommodation, forming the basis of our argument for resilient phenotypes.

### 4.1 Free energy and active inference: formalism

Having a generative model that entails agency, necessarily, postulates a model of the consequences of action in any domain (i.e., motoric or autonomic). In a minimal sense, this kind of generative model—under which the degree of surprise or free energy is defined—is a model of the self as agent.

To reframe, the brain essentially acts as a prediction-processing network, where prior beliefs are updated with new information to form posterior beliefs, in the spirit of Bayesian belief updating^3^ (Friston, 2010; Friston et al., 2006). Surprise cannot be quantified but is analogous to the variational free energy that systems seek to minimize. Beliefs are updated based on their accuracy, or the degree to which they correctly predict current sensory information, and their precision or reliability over time^4^ (Friston, 2009). Precision is encoded by neurons reporting prediction errors being given higher synaptic gain—encoded at fast timescales through neuromodulation of synaptic efficacy, and through to neuroendocrine-mediated plasticity and learning, and slower structure learning and reconfiguration of generative models associated with changes in immune responses (Arnaldo et al., 2022).

Technically, EFE, i.e., expected surprise, is reduced through action via policy selection (Arnaldo et al., 2022). Acting to optimize preferred sensory inputs will pre-emptively minimize the divergence between anticipated and sampled sensory input, thereby minimizing expected surprise (Friston et al., 2016). Expected surprise can also be reduced by updating relevant beliefs to reduce uncertainty. These two aspects of minimizing EFE can be read as epistemic and instrumental or pragmatic affordances; in exactly the same way that distinguishes between exploration and exploitation. In other words, the single imperative to minimize EFE manifests as curious, information-seeking behavior that is constrained by the prior preferences that shape goal-seeking behavior. The relative precision of epistemic and instrumental affordances translates into preferences to explore or exploit the environment (Caddick and Rottman, 2021; Friston et al., 2017), and may rest on genetic, epigenetic, and developmental factors.

It is also helpful to decompose EFE into risk, the difference between predicted and *a priori* preferred outcomes in the future, and ambiguity, which is the uncertainty associated with future observations, given current states (Da Costa et al., 2020). This further clarifies the role of exploitative, i.e., risk minimizing (goal-seeking), and explorative (information-seeking), ambiguity minimizing, policy selection. Da Costa et al. (2020) highlight the crucial role of awareness as such: “planning and decision-making, respectively, correspond to evaluating the expected free energy of different policies, which scores their goodness in relation to prior preferences and forming approximate posterior beliefs about policies.” This EFE formulation of active inference has also been related to interoceptive control under allostasis (Tschantz et al., 2022), supporting the idea of allostatic growth, using the same model as AL.

The enactive process described by Friston et al. (2021) as sophisticated inference is also essential to our model. Using the economic definition of sophisticated as ‘beliefs about beliefs’, or meta-beliefs, of either one’s own or others, meta-beliefs are stable, high-level beliefs that constrain lower-level beliefs—are foundational for the enactive niche construction. Meta-beliefs begin with the assumption that the intrinsic value of every action is its epistemic value or affordance (Friston et al., 2015). Under sophisticated inference, planning becomes a belief generation strategy. This link introduces conditional dependencies between the past for the selection of actions in the present and for similar selection of future paths, e.g., mental time-travel (Friston et al., 2021). In effect, these meta beliefs reflect beliefs states or “what I would *believe* about what would happen if I did that,” as compared to “what would happen if I did that” as is the case when modeling a single belief (Friston et al., 2021).

### 4.2 Awareness under active inference

Impacting the successful formation of predictions, we consider three interacting levels of awareness as consistent with the multidisciplinary literature on cognition and psychology and easily integrated into active inference: (1) sensory, (2) interoceptive, and (3) enactive, or meta, awareness. We observe overlap in these levels similar to the heterarchy, Arnaldo et al. (2022) propose to account for the coding of precision of beliefs across timescales. These levels of awareness similarly occur across timescales and can feed forward and backward to impact awareness in the other levels.

Sensory awareness and processing relate to the rapid neuromodulatory and neurotransmitter effects of sensory inputs; in subjective terms, what we are hearing, seeing, feeling, smelling, and tasting at each given moment. Sensory differences are implicated in numerous clinical conditions (Harrison et al., 2019). Sensory awareness predominates in certain enactive strategies, where sensory sensitivity and processing disruptions interrupt the development of enactive awareness as in obsessive-compulsive disorder (OCD) (Hulle et al., 2019). Sensory awareness precedes appraisal in our formulation. Conversely, we argue that when enactive awareness is developed through optimization of the enactive niche, sensory awareness provides input into all predictive models including epistemic models of self as well deep temporal “planning as inference” models.

With heightened sensory sensitivity, overly precise beliefs can be developed that override prior beliefs that underwrite intentions and agency, resulting in false perceptual inference, e.g., delusions in schizophrenia (Friston et al., 2013) or an awareness-enhanced level of precision associated with maintenance of negative emotional states (Schwartenbeck et al., 2015).

Interoceptive sensations constitute the afferent physiological information from the body to the brain and allow the organism to be aware of its regulatory status. Interoceptive awareness provides feedback on the success of enactive allostasis as inputs provided by the viscera (Craig, 2003; Quadt et al., 2022) and, in combination with sensory awareness and ANS and HPA activation (Schulz and Vögele, 2015). Providing mental percepts for afferent internal inputs is critical to the construction of affective and social experience (Barrett et al., 2016; Quigley et al., 2021; Seth, 2013). Interoception may influence “the dynamic basis to the concept of self” (Critchley and Harrison, 2013). These levels of interoceptive awareness also operate under active inference to minimize unexpected, energy-consuming surprises (Paulus et al., 2019). Thus, interoception may not only impact, or possibly be, awareness (Seth, 2013); it is also critical in the ability to understand others’ intentional and belief states, i.e., theory of mind (Ondobaka et al., 2017). Social allostasis extends similar mechanisms to group dynamics (Saxbe et al., 2020).

Finally, enactive awareness encompasses what others have described as self-awareness (Friston K., 2018) or meta-cognition (Fleming and Dolan, 2012), but also includes a nested awareness of how one’s actions impact the experience—and construction—of one’s environment, again part of the enactive niche. This brings to the fore the importance of belief-based behavioral policy selection within an enactive niche. Behavioral policies are selected to optimize EFE, which requires epistemic (exploration) and pragmatic (exploitation) behaviors to reduce uncertainty and to enable reward-seeking, respectively (Friston et al., 2016) within the enactive niche. At the highest level, enactive behavioral policy selection requires sophisticated inference, or nested beliefs about beliefs (Friston et al., 2021), which can each be optimized. Because sensory and interoceptive errors feed into enactive awareness, the latter includes perception and emotion, but also includes higher-order “top-down” processes. This allows for direction of attention that impact those processes, including cognition, mental time travel, the construction of concepts of self and self-esteem, and the construction of social representations necessary for empathy and compassion. Deane et al. (2024) refer to adaptive narrative control or the ability to model one’s own attentional states and how they can be controlled as the mental action that allows for affective and physiological regulation. It is sophisticated inference that allows for the proactive development of enactive awareness and policy selection capable of reducing EFE in one’s niches, potentially extending to novel, even chaotic, enactive niches.

Within enactive awareness, we include default states or biases which stem from a combination of genetics, epigenetics, and experience, which can influence one’s beliefs without overt awareness. In addition to experience, each organism is imbued with some degree of evolutionary determined preferences that underwrite precise constraints on enactive beliefs and policies. Be it explorative or exploitive, inhibitory or appetitive, there are core preferences satisfying evolutionary demands that influence phenotypic predictive processes. Although many predictive models operate independent of awareness, e.g., osmotic adjustments and circadian hormonal fluctuations, there are also beliefs and policies that through a variety of genetic, epigenetic, and learned mechanisms underwrite beliefs that inferentially select policies that may or may not use awareness. When such beliefs select behavioral policies without the influence of awareness, it reflects bias, or the default state of predictive processing.

When sensory attention (i.e., awareness) is decreased, we are more likely to rely on prior beliefs (i.e., inductive biases) for policy selection. These include such innate policy selections as those imbued by personality preferences or learned habits, such as placing one’s keys in the same place each time—although this may remain aspirational for some. Regardless, biases can be adaptive or maladaptive. If maladaptive, as so often is the ubiquitous confirmation bias (Tversky and Kahneman, 1974), they can only be altered with effortful, top-down attention.

Predictive processing in the absence of awareness can reduce expected surprise in many situations and informs how habits develop and are maintained. Such biases can also serve to perpetuate uncertainty, a process that may explain associations between personality and mental health. Traits associated with low extraversion and high neuroticism (Hakulinen et al., 2015) can be argued to reflect a lack of emotional regulation and a sense of self indicative of a bias that tolerates expected uncertainty (Clark et al., 2018).

Stress directs cognitive resources to limbic vigilance, decreasing cognitive attention and flexibility. Stress amplifies bias leading to preference for habitual, overlearned solutions without exploring new options. We refer to this as belief bias, at least partially distinct from the more common emotional and cognitive biases. Belief biases are rarely context specific and reflect meta-beliefs that are not fully enactive, i.e., do not minimize EFE. Belief biases act as the lens through which we process the world, e.g., evolved control parameters associated with personality (Safron and Sheikhbahaee, 2023). When used in model generation, (meta) belief bias fails to select policies that consider the full repertoire of coping or responses—and restrict optimally enactive policy selection. Such biases as personality develop from characteristic adaptations and evolutionary selection to play a role in long-term phenotypic plasticity and adaptation and may also play a role in canalization.

### 4.3 Resilience as predictive allostasis

Reflecting the predictive link shared between allostasis and active inference, efforts to frame allostatic resilience were previewed by Feldman-Barrett and colleagues who presented depression as an allostatic disruption (Barrett et al., 2016). This work identified interoception as the signal of allostatic change, arguing that if allostasis is central to brain architecture, affect is better considered as an aspect of consciousness, not emotion *per se*, and that all perception is a consequence of predictive allostatic change represented through interoception. In our terminology, these are beliefs, which in the case of depression, result in prediction error causing repeated negative interoceptive signals. Over time, the net result is a metabolically and statistically inefficient internal (i.e., generative) model.

Miller et al. (2022) suggest three concepts of resilience; (1) inertia or the state of being resistant to change, (2) elasticity or bouncing back to its setpoints, and (3) plasticity or the ability to expand one’s repertoire of good states. Under an active inference model, inertia is argued to reflect high precision of prior beliefs, although the potential for inflexibility to develop is recognized. Elasticity is related to homeostasis and allostasis, and the use of temporal models to anticipate the consequences of future actions allows for recovery from environmental or prosocial perturbations. The notion of optimality is discussed in terms of balance between epistemic and instrumental actions, with resilience requiring the cognitive flexibility needed to explore new hypotheses and then update models in terms of updating the relative precision of prior preferences (i.e., biases) that, in turn, affect the balance between epistemic and instrumental affordances. Finally, plasticity is discussed in terms of degeneracy, or useful redundancy. While redundant systems are less efficient, degenerate models afford the opportunity to seek out surprises that are likely to provide maximal information gain. The argument is made that the best strategy is not to attempt to avoid inevitable expected surprises but to become a system that thrives amongst varying degrees of risk and uncertainty.

Recent work (Waugh and Sali, 2023) explicitly placed resilience and allostasis into an active inference framework. Linking resilience with emotional intelligence, they argue active inference selects for the maintenance of well-being. Defining resilience as a pre-determined ability that can be learned across life, a hierarchical tradeoff between belief development and updating is proposed such that a positive sense of well-being is at the top level of beliefs. Model stability is argued to reduce variational free energy as an explanation for why resilience persists through both minor and major prediction errors, with an exception being made for consistent minor errors.

## 5 The enactive niche

The construct of niche has been applied to active inference in several contexts (Bruineberg et al., 2018; Constant et al., 2022; Miller et al., 2022; Sladky et al., 2023; Tschantz et al., 2020). The cognitive niche was defined as a co-constructed ‘common ground’ that optimizes belief updating, also referred to as extended active inference (Constant et al., 2022). This formulation of niche captures the essence of our concept of enactive niche in intent, but we see an enactive niche as the sum total of all the sensory inputs, including interoception, against which the success of policy selection is evaluated. Policy selection within the enactive niche is based on posterior beliefs about the most plausible policy to pursue under the prior belief that will minimize EFE. Both prior and posterior beliefs include all unaware and aware mechanisms capable of reducing EFE within the given enactive niche. Within this enactive niche, we highlight the inseparability of our environment, our exchange with the environment, our allostatic systems, and our capacity for active inference. We stress the enactive role of situational awareness in the niche, impacting its own updating, as described in the cognitive niche, but also impacting policy selection.

We emphasize three elements of active inference in the setting of an enactive niche. First, (situational) awareness is both cause and consequence of predictive processing, thus framing thoughts as constructed by—and capable of influencing—predictions. This is essential in phenotypic plasticity as seen among individuals who accommodate a shift in the enactive niche by changing awareness. This is consistent with the emphasis on flexibility mindset in resilience (Bonanno et al., 2024). This increase in accommodation stands in contrast to the reactivity seen when a phenotype is ill-suited for the dynamics of their niche.

To put it vernacularly, our awareness can change our niche, and our niche changes our awareness; thoughts matter in the enactive niche. We do not find this to be a trivial issue in our formulation of resilience. Awareness, built on our uncertainty about future policy selections, is the inferential selection of policies that improve our ability to select future policies that minimize EFE. The niche also supports the development of meta-beliefs allowing for planning, both forward and backward, as inference, i.e., deep temporal models and cognitive flexibility, to reduce EFE.

The second element we emphasize is the role of awareness in interacting with the enactive niche. It is our awareness that allows for the possibility of reducing expected uncertainty across the entire niche. Engagement with the enactive niche using deep temporal models allows for proactive engagement with the entire niche. Social allostasis (Saxbe et al., 2020) suggests mechanisms for the bi-directional effects that relationships have on the individual. Because one expects to engage with multiple social groups and individuals in one’s daily social interactions and roles, one’s holistic social niche can be argued to be comprised of multiple partially overlapping niches. Enactive predictions may be systematically more or less accurate in these different exchanges; for example, interaction with family leading to predictions that result in complete allostatic accommodation than interaction in the workplace. Through aware beliefs such as compassion and gratitude, group homeostasis increases, thus decreasing the need for individual allostatic responses and promoting recovery when individual allostatic responses do occur.

This same level of engagement happens with the physical environment as the sensory information provided by the environment is processed for consistency with beliefs about the environment. History provides numerous models of how the environment exists in individual enactive niches, from the symbiotic niches of hunter-gathers to the domineering niche of industrialism to the digital niche of technology. Environments alter the entire niche including individual awareness. The clear increase in allostatic disorders that have occurred since industrialism speaks to a negative effect of aspects of this environmental change on allostatic predictions.

The final element we see as foundational to the enactive niche is the ability to construct an awareness that allows individuals to resist stress, i.e., to accommodate the chaos of new and/or changing enactive niches. Self-organizing agents are defined by their ability to develop, to some extent, enactive niches that defy entropic dissipation and dispersion. Humans have a unique ability to expand their individual and collective enactive niches into entropic environments. From an enactive niche perspective, this requires awareness and policy selection to act in coordination to switch between openness to chaos, i.e., being *pro-entropic,* intentionally embracing and enacting change in the niche, to a closed system when the individual resists further perturbations. This process of moving from open to closed systems, to greater or lesser degrees, underlies decision-making.

**Figure 1:**
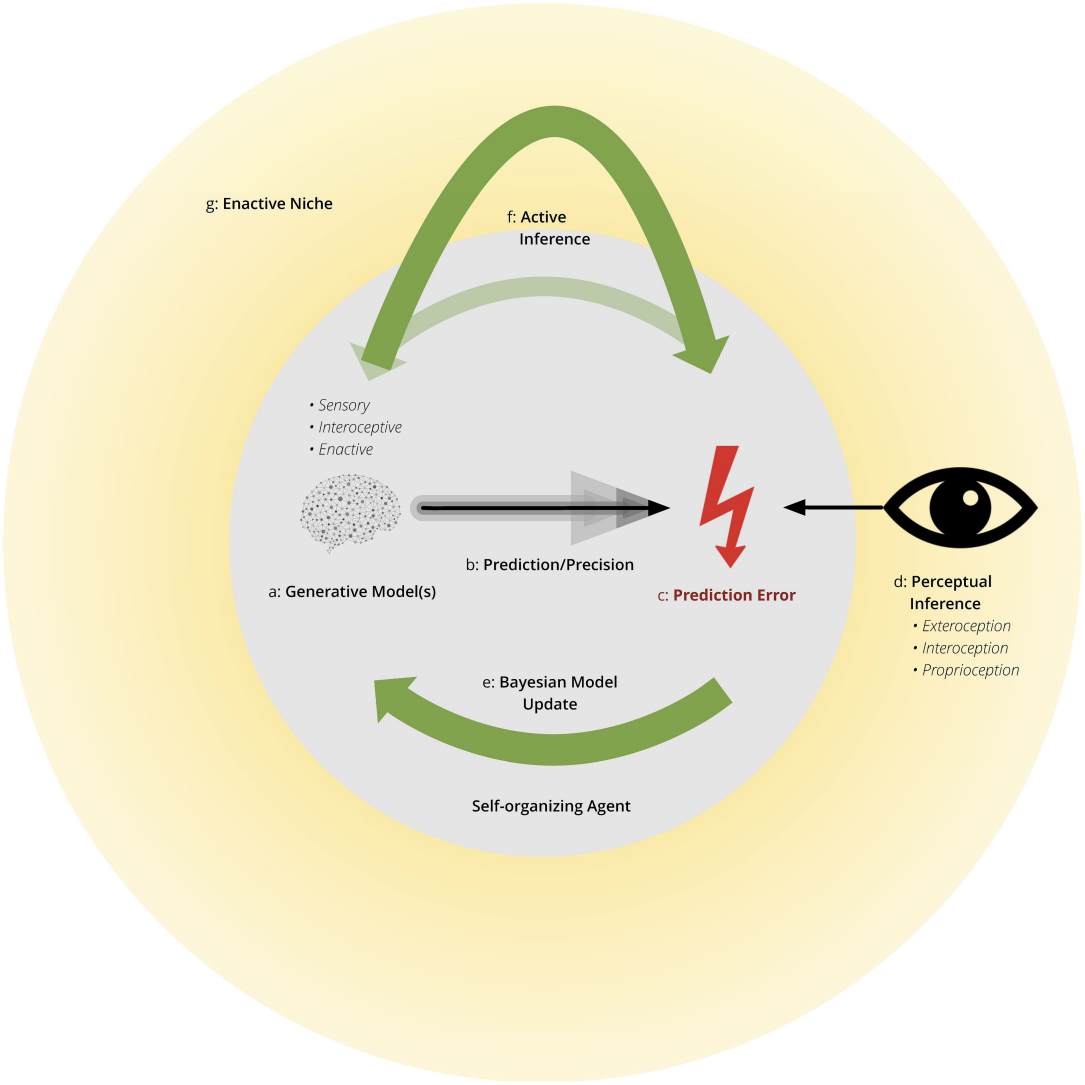
Self-organizing agent within an enactive niche. This conceptualizes the brain, operating as a self-organizing agent, within an entropic environment. In the process of self-organization, the agent interacts with the environment, with the potential of shaping the environment, as the environment simultaneously shapes the agent’s behavioral interactions. This forms an enactive niche. Specific elements include: *Generative Model(s)* are probabilistic models of the cause-effect structure of the environment. They generate predictions of incoming sensory inputs using relevant prior models engendered by genetic, epigenetic, development, and previous experiences. These Bayesian priors are adjusted by active inference to reduce (precision-weighted) prediction error, or surprise. These models generate predictions in all sensed modalities (i.e., exteroceptive and interoceptive) and, in deep or hierarchical predictions of predictions of precision (c.f., metacognition and attention, respectively) necessary for awareness. *Prediction Error* is the difference between predicted and sensory inputs and is synonymous with surprise. Mathematically, free energy is a computable upper bound on surprise. *Precision* scores the reliability, confidence or efficacy afforded predictions and prediction errors. Higher sensory *precision*, as indicated by darker lines, results in predictions with less tolerance for sensory error, leading to greater belief updating in the face of precise sensory information. *Bayesian Model (a.k.a., belief) Updating* uses precision weighted prediction errors to revise or update prior Bayesian beliefs into posterior beliefs (i.e., after seeing sensory input). *Perceptual Inference* provides the “best explanation” for the causes of sensory input by which predictions of sensory input enable prediction errors to update prior beliefs. Perception is part of active inference and includes exteroception, interoception, and proprioception. *Active Inference* selects policies to change the enactive niche to better align with predictions. As indicated by the lower arrow, active inference can also act upon awareness, effectively linking the environment and awareness. This results in an *enactive niche* linking the agent with all environmental and social elements with which the agent interacts. Active inference allows for the niche to shape the agent and the agent to shape the niche.

**Figure 2:**
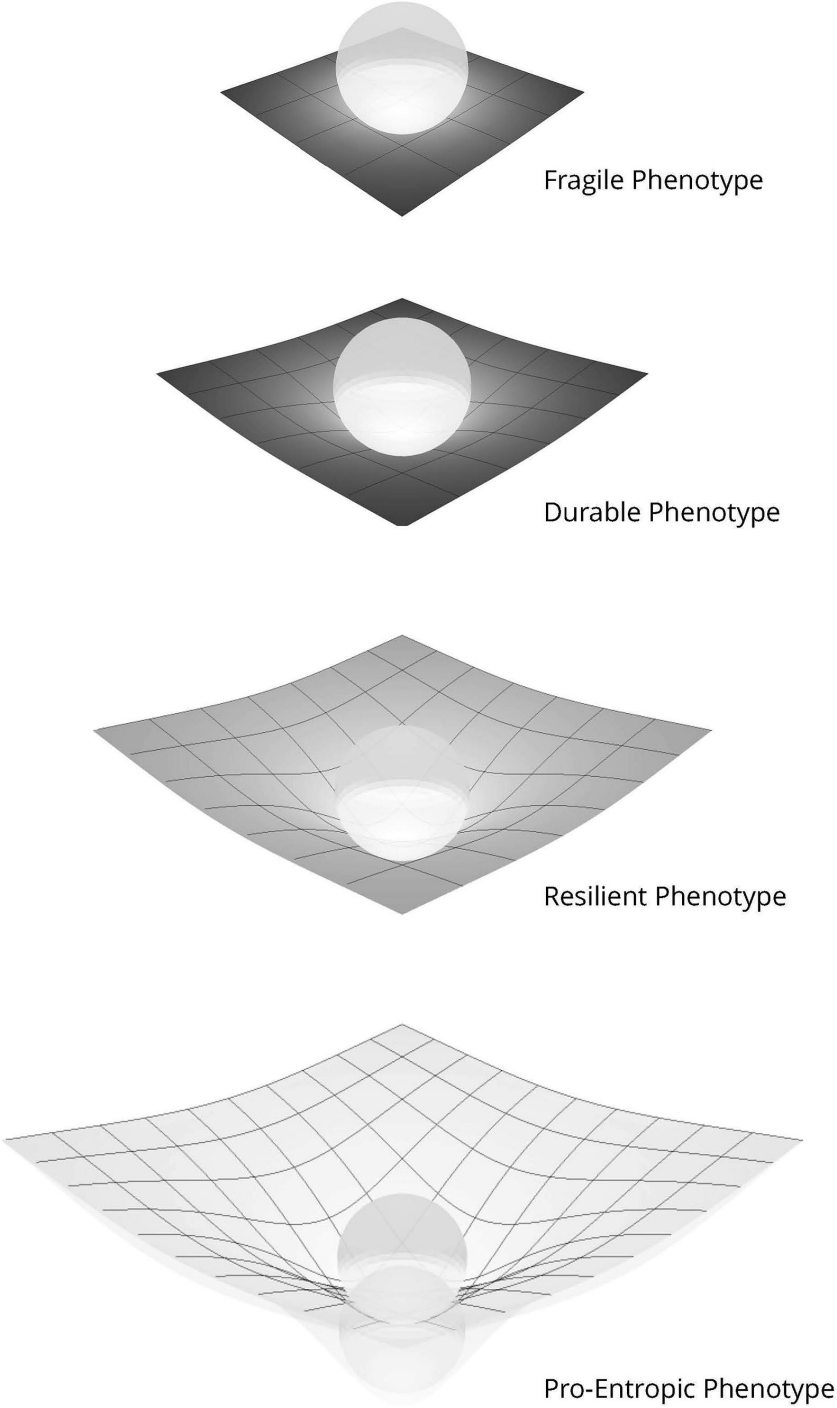
Schematic phenotypic enactive strategies. These schematics depict the relationship between the agent, as represented by the orb, and their enactive niche reflected by the grid. Three aspects of the grid are relevant: (1) the size: reflecting the range of physical and social diversity within the agent’s niche, (2) the shade: indicating the characteristic prediction error within the niche, lighter less error, and (3) the depth of the orb within the grid indicating the degree to which the agent has enactively shaped the niche. *Fragile* exists within a small niche with a limited range of accurate predictions. It exerts minimal influence on its enactive niche, rather exists as a slave to its senses. *Durable* has a well-defined niche within which it makes accurate predictions. Within this narrow niche it can shape the environment, provided the niche forwards minimal surprise. *Resilient* exists within a broad, diverse niche with a wide range of accurate predictions and an ability to accommodate surprise. It shapes its niche to better support its predictive allostasis. *Pro-entropic* has the characteristics of the Resilient with the addition of proactive enactive awareness; allowing context-sensitive and adaptive predictions even in novel niches, as suggested by the superimposition of the orb.

Enactive allostasis requires adapting to one’s niche. To be clear one’s enactive niche is dynamic, requiring constant updating of all model and policy distributions (see Figure 1 for a high-level overview of active inference within an enactive niche). Niches differ in the allostatic demands placed upon an individual, such that individuals may present themselves as resilient within certain social and physical environments but not others. Meanwhile, individuals vary in their ability to shape the dynamics of their niche in accordance with allostatic demands. However, these two factors highlight the variation in ways to achieve adaptation. Accounting for these two factors—the allostatic demands of the niche and the capability of the individual to shape their niche—the resilience phenotypes schematically presented in Figure 2 represent different strategies an individual may employ to successfully adapt within their niche.

These proposed phenotypes were presaged by the work of Bonanno (2004) and Bonanno et al. (2024) as outcome trajectories following exposure to potentially traumatic events. We find support in these trajectories for our central understanding of resilience as an allostatic phenotype that emerges within one’s current enactive niche. Approaching these differences from a free energy/enactive niche perspective allows for a formal theory of neural dynamics across spatiotemporal scales (Badcock et al., 2019).

Within this framework, we present descriptions of four proposed resilience phenotypes employing differential strategies using varying degrees of awareness and active inference, for variably active niche construction. More active construction relates to more successful accommodation of (variable) allostatic demand within the niche.

## 6 Resilience phenotypes

**Figure 3:**
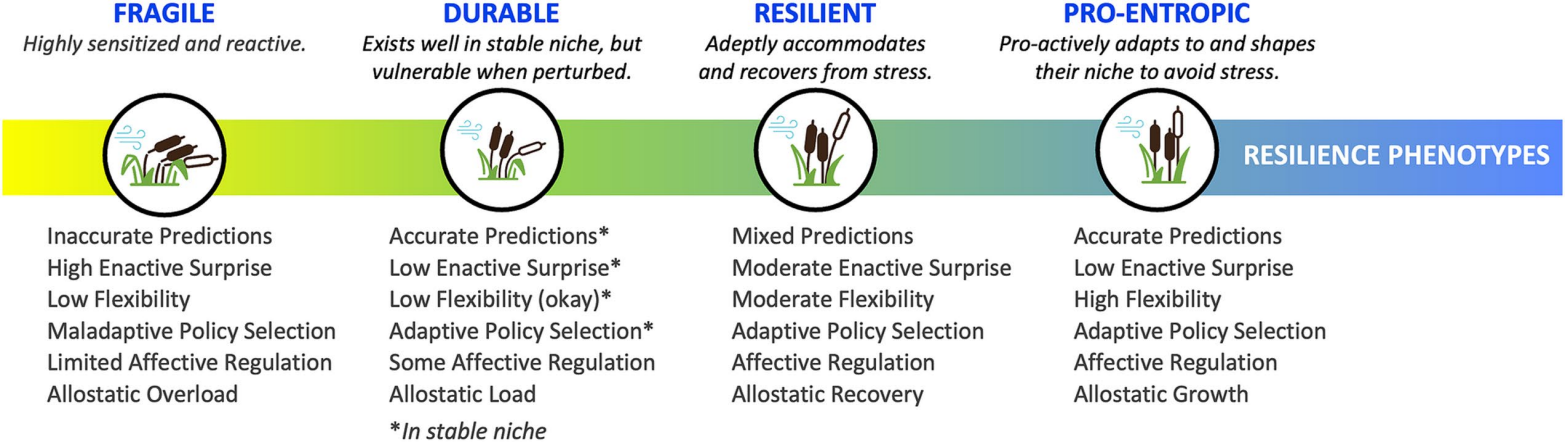
Continuum of resilience phenotypes. The four resilience phenotypes exist along a continuum—from fragile to durable to resilient to pro-entropic—and are characterized along key dimensions highlighted here and detailed in Table 1.

In our descriptions of each phenotype, we provide a brief operational definition, outline enactive strategies and awareness profiles and map them onto existing clinical categorizations. Table 1 provides an overview of this information, including preliminary clinical guidelines for phenotype identification and interventions. Figure 3 provides a visual representation of the continuum of phenotypes from fragile through to durable, resilient, and pro-entropic. Our goal is to provide a framework for the further study of enactive allostasis, including resilience, and resistance to stressful events, with emphasis on the role awareness may play in allostasis.

**Table 1:** Summary of resilience phenotypes.

	Fragile	Durable	Resilient	Pro-Entropic Resilient (PE)
Overview	Highly sensitized and reactive.	Exists well in stable niche, but vulnerable when perturbed.	Adeptly accommodates stress.	Pro-actively adapts to and shapes their niche to avoid stress.
Enactive strategy	Highly sensitized and often reactive and affronted by their environment. The focus on sensory inputs precludes the development of a positive epistemic memory.	They seek a predictable environment, and are surprised when it, or they, changes.	They are aware of and can modulate their stress response. They may be surprised when deeper models (sense of self, affiliative models) are violated.	Highly aware, they are attuned to sensory inputs and also have strong meta-awareness, allowing them to develop deep temporal models that avoid surprise in most cases. When surprised, they are aware of their ability to actively engage with their environment.
Allostatic profile	Constant surprise accumulates into allostatic (over)load, reflected in increased incidence of mental health diagnoses.	Allostatic load accumulates under surprise, reflected in increased reports of subjective stress.	Allostatic recovery; balanced counter-regulatory physiological reactions to challenge.	Allostatic growth; individual shows allostatic recovery following exposure to increased stressors and develop a risk tolerant system.
Flexibility	Low flexibility; presents as challenge: cognitive inflexibility engenders surprise; affective inflexibility reduces ability to regulate stress response; behavioral flexibility results in maladaptive policy selection.	Low flexibility, but little is needed within a narrow, stable niche.	With affective flexibility, can regulate and reappraise emotions. Some behavioral flexibility, borne of lower cognitive flexibility than the PE phenotype. For example, cognitive inflexibility may bias them to sub-optimal risk aversion.	Affective, cognitive, and behavioral flexibility, mediated by awareness and ability to switch between exploiting known solutions and exploring new ones as expected are beliefs violated.
Epistemic experience	Related to accumulated enactive surprise, they have a negative, unstable sense of self, poor affiliative processing, and tend to experience negative emotions and motivations.	One’s sense of self is primarily positive, but under-developed, especially a robust positive and flexible mindset and a flexible, supportive affiliative network.	Surprise is experienced, but surmounted, strengthening confidence in one’s own abilities (positive mindset) and/or recognition of stable sources of affiliative support (e.g., social connections, spirituality).	Borne of successful enactive adaptation, surprise is low and a stable, positive sense of self develops, which can engage in positive psychological constructs and affiliative behaviors allows for success during risk.
Clinical Guideline (combine self-report, behavioral observation, and available physiological measures)	Evaluate for heightened sensory sensitivity, sensory processing dysfunction, frequent allostatic reactivity, and limited cognitive/affective flexibility during stress	Evaluate for high vulnerability outside of routine settings, rigid beliefs tailored to specific niche, lack of interoceptive awareness.	Evaluate for effective allostatic recovery, interoceptive/ emotional awareness, with adequate cognitive flexibility and positive sense of self.	Evaluate for high awareness across sensory, interoceptive and meta levels, allostatic growth post-stress, and the use of inferential planning to facilitate proactive shaping
Interventions	Secure attachments. Pathological-specific treatment. Havening technique, Eye movement desensitization and reprocessing (EMDR), Safe and sound protocol	Mindfulness meditation, CBT with exposure to challenge rigid beliefs, Social skills training, Physical exercise in varied environments	Cognitive Behavioral Therapy with goal setting, Gratitude and compassion training, scenario-based planning exercises to develop deep temporal models and cognitive flexibility	Advanced mindfulness and self-compassion, Group-based problem solving, Episodic forward thinking.

### 6.1 Fragile phenotype

The fragile phenotype stems from genetic predispositions, developmental/epigenetic disruptions or overwhelming trauma, alone or in combination and presents as susceptible to psychological and physiological dysregulation. The commonality across these diverse etiologies and symptom clusters is ineffective allostatic predictions. Over time—and without change to the enactive niche—phenotypic canalization, or the opposite of phenotypic plasticity (Belsky and Pluess, 2013; Carhart-Harris et al., 2023), becomes increasingly entrenched, dominating the person’s cognitive, emotional, and behavioral experiences (Deane et al., 2024) and making allostatic predictions more difficult. Rigid and recurrent patterns persist despite allostatic errors that indicate a need for adaptation and change (Lewis and Todd, 2007). This suggests what has been termed “motivated inattention” or “avoidant mental action” (Deane et al., 2024) where reduced interoceptive awareness. i.e., lessened emotional recognition likely stemming from canalization, is a central mechanism underlying the fragile phenotype.

Many of the errors the fragile phenotype displays involve sensory processing disruptions, notably sensitivity to external and/or internal, i.e., interoceptive, sensory inputs, with frequent allostatic responses. Within a predictive framework, sensory precision and processing differences and stress reactivity impact awareness. With sensory disruptions the effect on awareness is to limit the ability of the person to use active inference to update their predictive models or enactive niche. The association of sensory reactivity and obsessive compulsive disorder (Ben-Sasson and Podoly, 2017), along with anxiety (Podoly and Ben-Sasson, 2020) supports the suggestion that sensory disruptions of the fragile phenotype may result in niche limitation.

Sensory differences, often overlooked in clinical research, are related to several mental health conditions (Harrison et al., 2019). Within a predictive processing framework, individual differences in sensory processing modulate attention given to sensory inputs (i.e., sensory sensitivity) and, crucially, the ability to ignore certain — usually self-generated — sensations (i.e., sensory attenuation). Regarding sensory awareness, assigning high precision to prior beliefs reduces the impact of incoming sensory prediction error signals, and vice versa (Clark et al., 2018).

The fragile phenotype may over-attend to specific external stimuli, thus updating beliefs with limited consideration of prior beliefs (Lawson et al., 2014). Failure to integrate sensory modalities is evident. Sensory over-reactivity has been associated with OCD and may reflect a high degree of uncertainty stemming from the persistently low precision of sensory predictions, with the obsessive need for order being a policy preference to mitigate sensory over reactivity (Poletti et al., 2023).

Like sensory awareness, interoceptive awareness may be altered, with a focus on a limited number of systems. This focus may at times play a role in the development of advanced specific interoceptive systems, e.g., high interoception is associated with an ability to detect others’ emotion (Dobrushina et al., 2020), and also with higher empathy (Fukushima et al., 2011). Impaired interoceptive ability has been reported in depression (Eggart et al., 2019) and autism (DuBois et al., 2016), while hyper-interoceptive sensitivity has been reported in anxiety (Domschke et al., 2010). Decreased interoceptive ability is related to elevated alexithymia (Brewer et al., 2016), which is associated with a range of disorders (Kojima, 2012).

The fragile phenotype demonstrates repeated allostatic responses, which promote even more frequent responses of longer duration and greater intensity (McEwen, 1998). Repeated exposure to rapid and strong allostatic responses without the foreseeable opportunity of recovery may result in allostatic overload (Fava et al., 2019). These conditions are colloquially referred to as burnout or exhaustion. This process of allostatic overload can be further understood as the top-down collapse of the “highest goals” (Goekoop and de Kleijn, 2021) similar to what is seen in hierarchical Bayesian control networks. As in sophisticated inference discussed above (Friston et al., 2021), organisms optimize to reduce the dimensions needed to most parsimoniously represent the niche. These higher level models interact with those lower on the hierarchy to use planning as inference to reach solutions and control behavior with a minimal amount of information, a formalization of Occam’s razor (Maisto et al., 2015). This hierarchical structure allows for the anticipation of more complex inter- and extra-personal events into deeper realms of the future using predictive modeling (Goekoop and de Kleijn, 2021). When an organism is confronted with conditions that interfere with the anticipated state, a useful definition of stress (Peters et al., 2017), prediction errors accumulate throughout the hierarchy, leading to the collapse of the highest level models, or allostatic overload. This top-down failure degrades integrative function at lower levels, consistent with the sensory and emotional focus of those suffering from states of overload such as burnout.

Exposure to severe and/or chronic allostatic activation during critical developmental periods has the potential to alter allostatic predictions across the lifespan (Wilkinson and Goodyer, 2011). Developmental exposure to trauma increases allostatic reactivity and sensitivity across the lifespan, increasing risk for not only stress-related disorders such as PTSD, but also for the entire spectrum of disorders of allostatic systems, e.g., metabolic, cardiovascular, immune (Danese and McEwen, 2012; Finlay et al., 2022).

Awareness in the fragile phenotype is aligned closely with sensory inputs. An inability to integrate sensory information is associated with AL (Azizi et al., 2024) and conforms with this phenotype. This has been alluded to as being “a slave to one’s senses” (Lawson et al., 2014; Peters et al., 2017; Quattrocki and Friston, 2014). With increased uncertainty, allostatic predictions are more closely linked to immediate neuromodulation of sensory inputs (Arnaldo et al., 2022). Given sensory over-reactivity, and focal interoceptive awareness, beliefs emphasize prediction of limited aspects of the environment. This circumscribes the ability to incorporate new experiences into beliefs. Beliefs in general are not updated quickly or with accuracy or precision. The inferential development of higher levels of awareness such as flexibility, theory of mind, and gratitude is inconsistent. Associated clinical characterizations include autism spectrum, OCD, affective conditions, Cluster B personalities, a history of ACES, and PTSD.

While this phenotype may be explained as allostatic over-reactivity stemming from childhood trauma and stress, it can also emerge from highly protected early environments. Notably, this phenotype can be expressed among individuals who were not previously fragile through a combination of prior experiences and current stressors developing an overly sensitive biological system, as can be the case in allostatic (over) load (Pfaltz and Schnyder, 2023).

When considered from the perspective of enactive allostasis, all aspects of the niche can be contributors to plasticity and to canalization. While we have focused on the maladaptive predictions characteristic of the fragile phenotype, it is important to consider interventions using this model. Specific interventions have been developed for a variety of conditions we understand to be part of this phenotype.

Havening, a technique using touch to develop adaptive processing of distressing thoughts and memories (Sumich et al., 2022), can be seen as using sensory inputs (soothing touch) to minimize sensory prediction errors to alter maladaptive prediction errors stemming from trauma and stress. Eye movement desensitization and reprocessing (EMDR) therapy assists patients through bilateral stimulation (Hase, 2021), usually guided eye movements, to reprocess traumatic memories by arguably reducing the prediction error associated with the trauma. The Safe and Sound Protocol, based on Polyvagal Theory (Porges, 2022), employs music tuned to the frequency of human speech to reduce auditory sensitivity and improve speech processing and social awareness in autism spectrum disorder (Kawai et al., 2023). The success of these and other sensory-based therapeutic approaches in improving adaptive functioning by focusing on sensory predictions which in turn enhance predictions across the enactive niche—support the existence of the fragile phenotype and the potential value of our model in identifying and developing interventions to enhance phenotypic plasticity.

### 6.2 Durable phenotype

This phenotype is reflective of a degree of canalization, but as opposed to the fragile phenotype, canalization is not necessarily associated with poor allostatic predictions. Carhart-Harris et al. (2023) has suggested four issues be addressed to understand the adaptivity of canalization: (1) the nature of the canalized phenotype; (2) the extent of canalization; (3) the initial context when the process of canalization began; and (4) any changes in context. We propose that the durable phenotype shows a high degree of canalization that can be adaptive under certain circumstances; for example, when a person lives within the strictures of a supportive religious community. The durable phenotype is most concordant with an organism preferring exploitation from genetic, epigenetic, and developmental sources; its nature in effect. The extent of canalization depends on the fit between the phenotypic nature of the organism and on the extent of boundary limitations of the niche. In an initial context—where the niche is well-suited to the nature of the organism—canalization develops efficiently for the specific niche. In this scenario, hierarchical higher order models are consistent across levels allowing for precise predictions and planning as inference within the niche. There is little need for accommodation within such a predictable niche thus canalization is efficient.

The durable phenotype often builds effective predictive allostatic response systems by living in defined cognitive and environmental niches (Constant et al., 2018, 2022). Within a predictable environment, durable persons experience little uncertainty and manage minimal surprise well. Active inference selects epistemic awareness to function within the social/environmental niche of the organism, which comports with the development of “echo-chambers” (Albarracin et al., 2022) or limited enactive niches. If the fragile type were to be described as a slave to one’s senses, the durable type could be considered slave to one’s rigid or narrow enactive niche. Epistemic awareness inferentially selects for the sense of self that reduces surprise within the enactive system. Growing up in Malibu versus rural Oklahoma has marked effects on the sense of self for the durable; “I’m a surfer,” “I’m a cowboy.”

The defined range of sensory inputs allows for the development of precise beliefs with high certainty, making it likely durable individuals will be unaware of the limited range within which the beliefs are accurate. This emphasis on precision, or bad bootstrapping (Miller et al., 2022), can result in predictive allostatic errors related to inflexibility as well as a tendency to develop fixed future positions. Provided that the environment is stable and basic biological and social needs are status quo, there is limited interoceptive awareness. This is a central feature of this phenotype. However, with a change to the niche, or what Carhart-Harris et al. (2023) call context, predictions made from canalization are rendered ineffective and allostatic errors made. This is analogous to the collapse of higher order models that result in the disintegration of lower order models moving the individual towards a more fragile phenotype.

In the durable phenotype, meta-beliefs, or beliefs about beliefs—for example estimates of the precision of prior beliefs—are updated for the niche within which the durable lives. Given the limited cognitive niche of the durable phenotype, awareness is not determined so much by sensory over-reactivity but by the demands of the niche. To maintain homeostasis, beliefs may conform with cultural systems that differ greatly from other niches, or from society in general, yet are functional for the individual’s allostatic system on a short-term basis. Without exposure to a wide range of enactive models, the ability to develop flexible epistemic awareness is hindered.

Even within one’s durable enactive niche, inflexibility can result in unexpected surprise and allostatic errors that accumulate over time, presenting as AL (Peters et al., 2017). We call attention to two conditions where the durable phenotype can be expected to be prone to ineffective allostatic predictions. If a person has genetic/epigenetic/developmental policy preferences (biases) that differ from their niche, uncertainty becomes likely and with time AL is accelerated. For example, if someone with a preference for exploration exists within a niche optimized for exploitation, their predictions will lack precision, making allostatic reactions more likely. Second, in the face of surprise, the repertoire of beliefs, meta-beliefs, and policies available to regain homeostasis is limited. If high levels of precision and certainty were developed within an enactive niche that becomes unviable or if the individual is seriously threatened in any way, allostatic overload is possible. Long-term, and possibly delayed, effects of allostatic overload are also possible, consistent with the delayed trajectory (Bonanno et al., 2011; Galatzer-Levy et al., 2018).

It is also possible that an individual developed beliefs within their enactive niche that allow for resilient responses after a large surprise or allostatic overload. The physical, cultural, and social context of the niche are of notable importance. Given the probabilistic nature of awareness under active inference, a narrow niche does equate with canalization. When a narrow niche underlies a degree of canalization that dominates individual awareness and constantly clashes with new situations and evidence, as well as wider societal norms and values, adversity is likely to increase fragility. Nevertheless, we *do* empirically observe incredible outcomes in individuals who have also faced incredible adversity—a phenomenon that has been known to make even the most die-hard empiricists feel some degree of awe at the human spirit—demonstrating the meta-awareness needed to explore new hypotheses and update models can emerge from very limited, even dysfunctional, niches.

**Figure 4:**
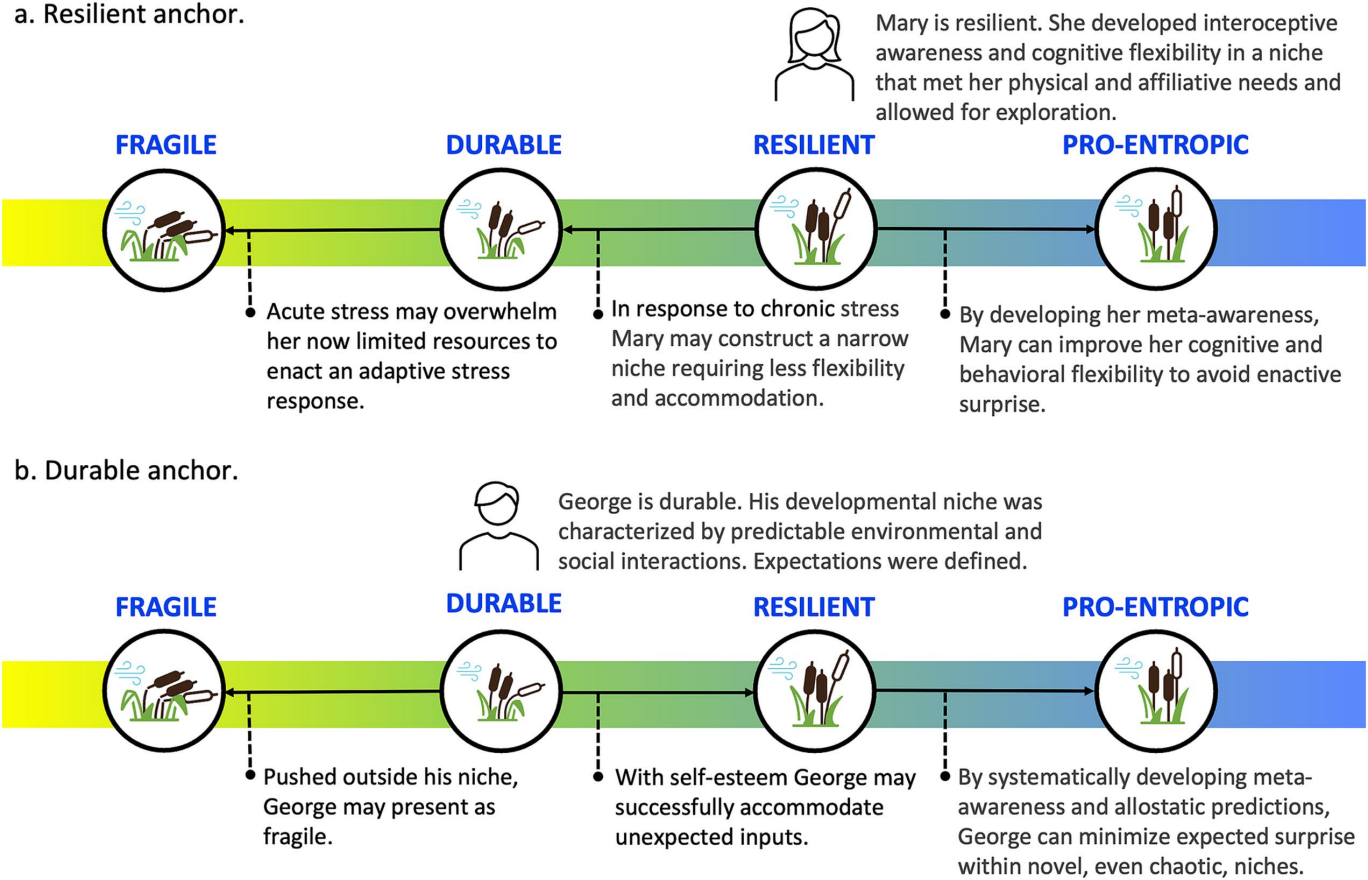
Vignettes of phenotypic plasticity. Highlighting the plasticity of the resilience phenotypes, we provide examples of two individuals’ current phenotypic presentation (“anchor”) and factors that can induce their movement along the resilience continuum. In these transitions, we emphasize the impact of different types of awareness on allostatic responses to changes in the enactive niche. **(a)** Mary presents as the resilient phenotype. **(b)** George serves as a durable example.

Cognitive flexibility helps in the application of prior models to new niches, explaining how the durable phenotype may move to successfully occupy a broader niche and/or actively shape the niche to fit their demands following a large surprise. This process could describe what is termed post-traumatic growth (Henson et al., 2021) and includes the development of a stronger sense of self, better social relationships, and a more grounded sense of purpose and meaning. Again, we see parallels to resilience trajectories here, specifically to that of recovery. Further we see the bi-directional nature of enactive niches, even when there are relatively constrained external resources, allowing for the possibility of positive phenotypic plasticity (see Figure 4 for vignettes of positive and negative phenotypic plasticity).

Again, we see therapeutic interventions being developed that support plasticity, in this instance for the durable phenotype. Specifically, those targeting interoception such as mindfulness meditation and the awareness and manipulation of breathing (Weng et al., 2021) have shown promise. Much as interoceptive sensations, e.g., increases in heart rate and respiration alter predictions toward emotional states such as fear and anxiety to reduce interoceptive prediction error, within enactive allostasis, awareness allows for immediate regulation of respiration, which alters interoceptive predictions away from fear. We see interoceptive awareness as a prerequisite for phenotypic plasticity in the durable phenotype.

### 6.3 Resilient phenotype

The resilient phenotype functions as durable within a broader enactive niche. With cognitive flexibility, allostatic systems consistently return to baseline after activation. The “elasticity” of this successful allostatic accommodation allows the enactive allostatic system to recover following exposure to a stressor; the regulatory and cognitive flexibility of the resilient phenotype can “bounce back” from stressors in the traditional sense. We also agree that the affiliation system is essential in the resilient phenotype (Darling Rasmussen et al., 2019; Feldman, 2020).

The defining feature of the resilient phenotype—from an enactive perspective—is the influence of interoceptive inference and a level of meta-awareness allowing for effective allostatic predictions outside of a predictable enactive niche. Unlike the durable phenotype, the resilient phenotype is consistently interoceptively aware, and generally emotionally regulated, enabling effective enactive allostasis. This interoceptive awareness allows for the development of temporal models needed to recover from stress in most situations.

Over time, successful allostatic accommodation engenders a positive sense of self. More specifically, there are cognitive processes that are consistent features of human enactive allostatic systems that return to baseline after challenge (Constant et al., 2022; Miller et al., 2022). A positive sense of self provides a consistent weighting for posterior beliefs and policy selection. The ability to think temporally allows the brain to simulate the outcomes of a variety of predictions prior to policy selection. Simulations are made with the current matrix of priors, allowing for adjustments based on the certainty of simulated outcomes, prior to an active inference.

Cognitive states change enactive allostasis. Cognitive appraisal of events can redefine them from a threat to a challenge, a redefinition that shifts the allostatic response from one with unknown accommodation to one of clear accommodation (Bobba-Alves et al., 2022). There is also evidence that states of awareness such as meditation, mindfulness, and awe have associations with allostasis (Klimes-Dougan et al., 2020). Physiological fitness and fit with the social/environmental niche are also foundational in this phenotype.

The elevated importance of awareness in this phenotype also highlights the role it can play in precluding resilience. Epistemic memories throughout hierarchical inferential processes do not occur to optimize resilience, or well-being as argued by Waugh and Sali (2023); they occur to minimize uncertainty and surprise. Rather than resilience being an ability or even a process, we see it as identifiable enactive allostatic predictions characterized by sensory and interoceptive awareness, cognitive flexibility and meta beliefs capable of accommodating allostatic surprise.

This enactive profile includes optimization of many aspects of the counteractive systems activated by allostasis. Without the benefit of empirical studies using measures that capture the dynamics of the enactive niche, current interventions developed to enhance resilience largely consider resilience traits and factors as outcome measure. Accepting these measurement limitations, a meta-analysis of training interventions including cognitive behavioral therapy (CBT) techniques, e.g., emotion regulation, goal setting, and mindfulness practices, e.g., cognitive flexibility, self-compassion, gratitude, found a moderate benefit over control (Joyce et al., 2018). The inclusion of metrics indicative of reactivity and accommodation—e.g., heart rate, heart rate variability, respiration, available on biometric devices—will expand understanding of the enactive niche and are a logical next step in resilience training.

The resilient phenotype has some ability to alter or migrate niches when the demands of the niche become unsupportive of their allostatic systems. If a resilient person finds their niche collapsing or shifting in ways that cause chronic stress, e.g., a workplace becomes toxic, niche change becomes an important component of this phenotype.

### 6.4 The pro-entropic (PE) phenotype

The pro-entropic phenotype emerges from the core principle that the brain reduces surprise through generative cycles of action and perception (Badcock et al., 2019; Friston et al., 2010). This has been rephrased by Badcock et al. (2019), (*ibid*) as, “every organism seeks to maximize sensory evidence for its own existence,” also termed self-evidencing (Hohwy, 2016). Implicit in this is a drive for self-actualization which in its complete expression defines the pro-entropic phenotype. When awareness and action act in harmony to not only accommodate surprise but also change some element of the niche to preclude future surprise, the pro-entropic phenotype emerges.

The PE phenotype assumes the enactive properties of the resilient phenotype with the addition that the allostatic system not only returns to baseline after activation but shows improved allostatic predictions after allostatic responses. At the other end of the spectrum from the fragile phenotype, we propose that sensory awareness is also high in the PE phenotype, but responsivity is appropriate, similar to the adaptive high-sensitivity profiles described for individuals raised in supportive environments (Nave et al., 2022).

Within enactive allostasis, much as certain predictive errors lead to allostatic load, aware engagement with enactive allostasis allows for proactive selection of interoceptive and exteroceptive sensory inputs. This can be thought of as aware enactive niche construction and enables not just allostatic recovery, as seen in the resilient phenotype, but allostatic growth, i.e., accommodation of increasing levels of stress exposure (both frequency and magnitude), both by more adept recovery, as well as heightened thresholds required to elicit an allostatic response. Heightened awareness as well as pro-active enactive shaping of one’s niche work together to increase one’s allostatic response threshold, including in novel social and physical environments.

We see PE as the Bayes optimal selection of meta-beliefs (and the resulting belief states). These belief states are selected in a recursive manner; this allows for the optimal selection of actions across any finite temporal horizon. Sophisticated inference allows for the identification of the end goal state and then through the use of backward planning through time, similar to game theory’s “backward induction,” to determine the course of actions that will optimally reach the goal (Da Costa et al., 2023).

Perhaps the defining feature of the PE is the ability to use sophisticated inference to proactively shape the external aspects of the enactive niche to fit with the regulatory needs of the person. Where the resilient phenotype that can adapt and “bounce back,” the pro-entropic phenotype proactively uses prediction to enact changes in their niche that minimize surprise. In this regard, much of human advancement can be seen as PE: we learned to control fire to survive in more fertile environments; much as we now develop business relationships that may be of future value. In addition to proactively shaping one’s enactive niche, the cognitive flexibility and temporal planning that are part of PE allow for the development of meta-models that can probabilistically infer the demands of new enactive niches. This allows for consideration of novel niches and more effective niche change when needed and/or beneficial.

PE also relies on the integrity of the underlying neurophysiological systems, sense of self, and temporal thinking. PE extends the role for awareness to include an understanding of the probabilistic nature of human life within the environment. Within this context, the PE phenotype proactively uses awareness to optimize active inferences that lead to preferred, unsurprising outcomes. Similar predictive processing models that describe directed awareness within a niche include, “when individual agents restructure their worlds so as to minimize internal processing costs and/or increase reliability,” (Constant et al., 2022) and as a “sense of our own poise over an action space” (Nave et al., 2022). It is this constructed enactive niche that affords PE and allostatic growth. Clinically we see this mapping onto active engagement with novelty, challenge, and creativity, while nourishing one’s niche and regulating well across new niches.

Interventions to enhance phenotypic plasticity may be as much informed by our history as by our present. When Aristotle argued that pain and adversity was needed for the drive for eudemonia, or self-awareness, personal growth, and ultimately contentment, he presaged much of the PE phenotype, as have generations of Buddhists and Hindus seeking enlightenment and dharma. However, we find in the model of enactive allostasis the potential for individualized strategies to facilitate the biopsychosocial operationalization of this wisdom of the ages.

A more pragmatic intervention with clear implications for PE has been receiving wider attention. Episodic future thinking (EFT) is the projection of the self into the future as a means of “pre-experiencing” an event (Atance and O’Neill, 2001; Schacter et al., 2017). This has been most widely studied in the context of delay-discounting, or the tendency to overvalue more immediate rewards. EFT has been repeatedly shown to be associated with a tendency to place higher value on delayed rewards with a concomitant improvement in decision-making (Rösch et al., 2022). This intervention has also been shown to be effective in treating disorders that relate to impulsivity, e.g., alcohol abuse (Myslowski et al., 2024). There are also suggestions that EFT may be an effective intervention to enhance resilient plasticity. Using both pilot work and literature review, Kent et al. (2015) conclude that making and manipulating internal models frees behavior from the present and allows it to become future-oriented. Episodic future thinking has also been associated with performance enhancement in such diverse dimensions as decision-making, emotion regulation, prospective memory and spatial navigation (Schacter et al., 2017). All these findings are consistent with active inference in general and the notion that interventions based on this theoretical model can enhance phenotypic plasticity toward PE. That cognitive rehearsal of future events increases the value placed on the future suggests that the higher order models that allow for inferential planning are strengthened by repetition. Any consistent method of strengthening higher order models is not only likely to improve the functioning of models lower in the hierarchy, such as the behavioral effects noted above, but also seem likely to allow for the higher order models to improve their robustness across novel situations, both of which we find characteristic of PE.

## 7 Discussion

Within the theoretical framework of enactive allostasis, we propose four resilience phenotypes: fragile, durable, resilient, and pro-entropic resilient. Within this model, individual differences in adaption to one’s environment can be predicted at various levels of inquiry; from genetics and epigenetics to counter regulatory physiological systems, to epistemic awareness and concomitant affective, cognitive, and behavioral flexibility.

In this enactive allostasis framework, the environment or enactive niche is inseparable from the individual, as is highlighted by the distinction between the fragile and PE phenotypes. Both are sensitive to the sensory inputs provided by their environment and body. These inputs are the central focus of the enactive niche within which the fragile exists, often leading to overly precise predictions without a clear reduction in uncertainty due to constrained sampling of sensory inputs. Consistent with an account of active versus passive coping best mitigating against physiological “wear and tear” experienced with stress and aging (Hawkley et al., 2005), with the PE sensory inputs are predicted within a broader enactive niche, allowing for a wider sampling strategy and utility of epistemic memory and deeper temporal models.

These cross-level characterizations speak to efforts to develop individualized or precision psychiatry (Friston, 2017). We propose that individuals with managed stress-related psychiatric diagnoses can express a durable resilience phenotype, which can switch back to fragile, depending on the success of their current niche adaptation. For example, while traditional resilience research examined binary risk versus protective factors for the development of PTSD (Yehuda, 2004), in our phenotypic niche adaptation framework, the same individual in one environment may be fragile and susceptible to intrusive thoughts, an exaggerated startle response, and maladaptive avoidant behavior. Conversely, in a more stable, predictable environment, they may not exhibit PTSD symptomatology and may adapt to thrive and express as resilient or PE phenotypes. It would be relevant if recent multi-domain hybrid analytics (categorize, cluster, classify) used to identify subtypes of PTSD following acute trauma exposure (Ben-Zion et al., 2020) support such phenotypic plasticity over time. In addition to adapting to the environment, management may require active updating of beliefs, for example that which is assisted by cognitive behavioral therapy or psychoanalytic insights. This is not to say that the fragile phenotype cannot engage in chaotic environments. Rather there is plasticity between the phenotypes; considering a constellation of measures across several levels of analysis allows one to make a probabilistic inference about one’s current phenotypic expression and whether an individual is likely to accommodate and recover from exposure to some degree of encountered challenge.

This is not to minimize the effect of the default state of the brain when making generative predictions. The degree to which genetic, epigenetic, developmental and previous experiential models influence predictions when the brain functions from its default, i.e., bias, state is foundational in understanding phenotypes and plasticity.

Extending the argument that resilience can be bolstered in individuals with strong bias states acquired from experiential sources, e.g., trauma, stress-related conditions such as PTSD, anxiety and depression, our enactive framework can also theoretically be applied to bolster resilience in neurodevelopmental conditions like autism and ADHD. Supported by early theoretical work (Sinha et al., 2014) and research on resilience in adults with autism (Ghanouni and Quirke, 2023), assisting such individuals develop strategies to shape and adapt to their environment over time offers a tractable perspective for fostering adaptability. In this framework, our resilience phenotypes, and others that might be described in the future, can move the field of resilience research away from investigating protective and risk factors for mental health diagnoses that reflect a crystalized view of resilience. Rather, one can be diagnosed with a psychiatric condition or developmental disorder at some point in one’s life and still exhibit an adaptable phenotype. Building on the sensory processing and interoceptive enhancement techniques mentioned above, the range of adaptive allostatic predictions can be systemically expanded.

In the enactive niche there are environments and states of awareness that improve allostatic predictions across all phenotypes. The value of exercise, positive relationships, time in nature, of living in tune with the basic circadian rhythms of life, are enactive choices that with repetition become engrained in our enactive niche. This strengthens homeostatic mechanisms and allows the individual to engage with stressful environments with less allostatic reactivity and raises the possibility of acting upon environments to reduce expected surprise. We view this level of enactive niche, with temporal models that reduce future uncertainty through proactive shaping of the environment, as most likely with a level of awareness constructed through prior positive social and environmental niches.

Mechanistically compatible with an enactive allostatic framework, this holistic approach is apt to foster generalizable adaptable resilience, so that the body’s stress response system can be co-opted to promote peak performance and well-being, shifting resilience research from avoiding “vulnerability” to mental health diagnoses, to increasing and growing an individual’s adaptability, shifting from solely pursuing precision psychiatry to a science of precision mental wellbeing.

This framework also holds implications for exceptional performance and the popular concept of growth mindset. From an enactive perspective, growth and exceptionality are accommodated by the niche, not the individual. While we have stressed the role of awareness in PE, this level of awareness, i.e., deep temporal modeling and flexibility, develops in the context of its niche. In considering individual resilience, the entirety of all biological, social and environmental systems that underwrite awareness create the enactive niche. Within this niche it is the inferential selection of adaptive policies that allows for the participation of the individual in phenotypic plasticity, in effect allowing for the construction of a resilient allostatic person with a niche of their own design.

We offer these phenotypes primarily as a point for further discussion, recognizing the complexity of the systems involved in discussing resilience from a model of an enactive niche. Numerous relevant points have been largely overlooked in this paper so as not to stray from its focus on predictive allostasis and the phenotypic framework. The impact of socio-environmental factors on allostasis is well-established and needs to be discussed in phenotype development. The full gamut of issues that influence phenotypic expression also warrants exploration, cultural issues foremost among this list. Methodological concerns also remain to be explored; factors influencing phenotypic stability versus plasticity over time and consideration of the clinical and analytic criteria to be used in defining boundaries between phenotypes.

Given the large amount of phenotypic variability and plasticity inherent in this model, advanced quantitative analytic techniques are needed to confirm the model. Such a measurement schema has been advanced as a principled Bayesian model of emotional valence (Hesp et al., 2021). Relying on the assumption that feeling good or bad, i.e., emotional valence, is critical to survival and is largely predictable using deep active inference to estimate overall model fitness. Understood at psychological, neuronal, behavioral and computational levels, second-order beliefs (beliefs about beliefs) track affective change. The concept of criticality, defined as the dynamic of persistent attractors between a stable and an unstable phase, has been suggested to be informative in understanding differences between such states as allostatic load and allostatic repair, i.e., resilience (Bettinger and Friston, 2023), and will be important as efforts to model resilient phenotypes and their plasticity proceed.

A few additional points we hope to further explore include the formalization of possible clinical guidelines and interventions to promote plasticity toward the PE phenotype. Possibility that both aware and unaware aspects of the phenotype may change across context. Given that phenotypic expression is evinced in an enactive niche, changing environments, either social or physical, may change the mechanisms underwriting awareness. For example, a person may be highly resilient and flexible in a fast-paced work environment yet express as fragile and inflexible when in the context of personal relationships. Such contextual phenotypes may suggest individual development in overlapping but distinct enactive niches. Extending this concept, social roles may evince different resilient phenotypic expressions and offer a target for future work.

## Data Availability

The original contributions presented in the study are included in the article/supplementary material, further inquiries can be directed to the corresponding author.

## Footnotes

^1^In all instances where we use the term “active inference,” “enactive inference” would also be appropriate.

^2^We recommend these comprehensive reviews for a broader discussion of the neurobiology of resilience (Kalisch et al., 2024; Osório et al., 2017; Russo et al., 2012).

^3^Technically, minimizing variational free energy is the same as maximizing Bayesian model evidence (a.k.a., marginal likelihood). This means that there is a mathematical isomorphism between minimizing surprise and maximizing the evidence for generative models – that is sometimes referred to as self-evidencing (Hohwy, 2016). When we talk about Bayesian beliefs, we refer to posterior probability densities encoded by neuronal activity and collectivity—as opposed to the propositional beliefs of folk psychology. In other words, Bayesian beliefs are a mathematical (subpersonal) construct.

^4^Prediction errors that are afforded more precision or confidence have a greater effect on posterior beliefs or belief updating, i.e., adjusting priors to explain away current prediction error, and by actively selecting sensory inputs to better conform with one’s beliefs. This belief updating process conforms well with active sensing and perceptual control theory (Parr and Friston, 2018).
